# Habits and Products of Germs

**Published:** 1898-04

**Authors:** Ernest B. Sangree

**Affiliations:** Professor of Bacteriology in the Medical Department of Vanderbilt University, Nashville, Tenn.


ARTICLE IV.

### HABITS AND PRODUCTS OF GERMS.

BY ERNEST B. SANGREE, A. M., M. D.

Professor of Bacteriology in the Medical Department of Vanderbilt University, Nashville, Tenn.

During the last few years the Brief has contained a number of articles dealing with the subject of bacteria and their products, but as all the articles seem to be on one side of the question, I thought it might not be out of place to have a few words on the other side. From the tone of some of the articles I gather that the authors believe bacteria to belong to the animal kingdom. This is a mistake. Bacteria are microscopic vegetable organisms. Then some writers assume that bacteriologists claim that all bacteria are harmful, and that most diseases are caused by germs. No bacteriologist makes any such claim. Out of the vast number of different kinds, only a few are known positively to be hurtful to man; most are innocuous, and many are beneficial. Bacteria differs from most other vegetation in that they have no green coloring matter, or chlorophyll, so universal in ordinary vegetation. By means of this chlorophyll, trees, plants, flowers, and the like, are able, in the presence of sunlight, to take carbon dioxide out of the air, and convert it into the carbon compounds making up their substance. Because of the want of this chlorophyll bacteria can not get carbon dioxide from the air, but must take it from organized carbon compounds. Most of them get their carbon from dead organic matter. These are called saprophytes, and they are our friends; for through their agency all dead things, animals, man, and trees are disintegrated, and the various elements resolved into forms that can once more be taken up by the bodies of living animals, men, and trees. Some bacteria can get their nutrition indifferently from living or from dead organic matter, and a few seem to be able to live off living organic substance alone. All such are called parasites. These bacteria can break up the cellular structures of the human or the animal body, and thus multiply at the expense of their host. They multiply with such increditable rapidity that, although each individual is extremely small, when the number mounts up into many millions and billions, the mass becomes very considerable. There is nothing strange in bacteria living off animal tissues. All nature is full of parasitism, of animals living off other animals, as illustrated by the various intestinal worms, and the varieties of lice; of animals living off plants, and the various forms of plant lice; of vegetation living off other vegetation, as the mistletoe and other parasitic plants; and finally of vegetation living off animal life, as the mould fungus that causes thrush, and the other well known fungi that cause barber's itch, fevers, and pityriasis versicolor. In this latter class belong the bacteria, which are much like the moulds in character, but which are generally much smaller, and multiply in a slightly different way.

All these parasites get their nourishment from some other organism that has already elaborated it, and frequently the parasite demands so much that the host has not enough left on which to live, and in consequence, dies. Much is said about the toxins or poisons made by bacteria, which are so hurtful to the person, or animal, infected, and about which some of your correspondents occasionally make merry. Bacteria are not the only vegetation that manufactures poisons, as every doctor well knows. Our most deadly drugs are produced by vegetation. Our strychnine, morphine, atropine, and hydrocyanic acid are all made by plants.

The nettle's sting will soon raise painful wheals, the juice of the Jequirity bean will cause a violent ophthalmia, the touch of the poison ivy will give rise to a vesicular dermatitis that will last for weeks. The deadly character of the poison from the pathogenic bacteria can easily be proven to anyone's own satisfaction, if he will perform an experiment like this, one that I have often made. Into a flask containing about a pint of sterile bouillon, put a few anthrax, diphtheria, typhoid, or tubercule bacilli. Let them multiply for a month, and then examine to see that the bouillon still contains but one kind of bacillus. Filter all the microbes out, and inject under the skin of a healthy rabbit, guinea pig, or rat, a little of the filtrate; at the same time injecting under the skin of another animal of the same kind a similar amount of sterile bouillon. Before the injection take the temperature of each animal, and continue doing so every half hour or hour. The cultures now contain so much toxin elaborated by the myriads of bacilli, that sometimes within a minute or two I have seen an animal go into convulsions, and die in five minutes. In half an hour I have seen a rat's tempetature drop from 101° F. to 95° F., and in an hour to 93° F. If the dose is not overwhelming, the animal may recover after showing every evidence of violent poisoning. Different animals succumb to different doses. The autopsy shows, in some cases, disintegration of the blood with escape of the hemoglobin, also, general cellular and necrotic changes, and evidences of distruction in the nerve and ganglia cells. The animal injected with bouillon remains unaffected. Or, try this: Take from a pure culture of anthrax bacilli, a very tiny amount, about half the size of a pin's head. Trim away the hair at the base of the tails of two brown mice, and snip through the skin with sterilized scissors. Now, rub under the skin of one mouse one half the anthrax culture taken, and after having killed, by means of heat or chemicals, the other half, rub it under the skin of the other mouse. Within six or eight hours the first mouse will sicken, and within a day, or a little over, it will die. In its blood and in all its organs will be found thousands upon thousands of the anthrax bacilli. The second mouse will be unaffected.

When one has repeated this experiment a number of times, it seems about as plain a case of cause and effect as when a hunter, spying a rabbit, shoots at it, sees it fall, finds it dead, and discovers in its dead body the shot that were previously in his gun.

The hunter is not apt to reason that the rabbit just happened to die at the moment he discharged his gun, and that the shot were probably born in the animal. But my article grows too long, and I must refrain from adding other simple proofs. I would suggest in closing, however, that if the excellent contributors to the Brief would do a little more work in the laboratory, with microscope, test-tube, and microbe, they would probably be a little less decided in their statements.—*The Medical Brief.*

